# Left Ventricular Twist and Circumferential Strain from MRI Tagging Predict Early Cardiovascular Disease in Duchenne Muscular Dystrophy

**DOI:** 10.3390/diagnostics15030326

**Published:** 2025-01-30

**Authors:** Zhan-Qiu Liu, Patrick Magrath, Nyasha G. Maforo, Michael Loecher, Holden H. Wu, Ashley Prosper, Pierangelo Renella, Nancy Halnon, Daniel B. Ennis

**Affiliations:** 1 Department of Radiology, Stanford University, Palo Alto, CA 94305, USA; mloecher@stanford.edu; 2 Cardiovascular Institute, Stanford University, Palo Alto, CA 94305, USA; 3 Department of Bioengineering, University of California, Los Angeles, CA 90095, USAholdenwu@mednet.ucla.edu (H.H.W.); 4 Department of Radiological Sciences, University of California, Los Angeles, CA 90095, USA; nyasha.maforo@pennmedicine.upenn.edu (N.G.M.); aprosper@mednet.ucla.edu (A.P.); prenella@choc.org (P.R.); 5 Physics and Biology in Medicine Interdepartmental Program, University of California, Los Angeles, CA 90095, USA; 6 Department of Medicine, Division of Pediatric Cardiology, CHOC Children’s Hospital, Orange, CA 92868, USA; 7 Department of Pediatrics, University of California, Los Angeles, CA 90095, USA; nhalnon@mednet.ucla.edu; 8 Maternal & Child Health Research Institute, Stanford University, Palo Alto, CA 94305, USA

**Keywords:** Duchenne Muscular Dystrophy, cardiomyopathy, cardiovascular magnetic resonance imaging, late gadolinium enhancement, strain imaging, MRI tagging, left ventricular ejection fraction, left ventricular circumferential strain, left ventricular twist, left ventricular torsion, left ventricular circumferential-longitudinal shear angle

## Abstract

**Background/Objectives:** Duchenne Muscular Dystrophy (DMD) is a prevalent fatal genetic disorder, and heart failure is the leading cause of mortality. Peak left ventricular (LV) circumferential strain (E_cc_), twist, and circumferential-longitudinal shear angle (θ_CL_) are promising biomarkers for the improved and early diagnosis of incipient heart failure. Our goals were as follows: 1) to characterize a spectrum of functional and rotational LV biomarkers in boys with DMD compared with healthy age-matched controls; and 2) to identify LV biomarkers of early cardiomyopathy in the absence of abnormal LVEF or LGE. **Methods:** Boys with DMD (*N* = 43) and age-matched healthy volunteers (*N* = 16) were prospectively enrolled and underwent a 3T CMR exam after obtaining informed consent. Breath-held MRI tagging was used to estimate left ventricular E_cc_ at the mid-ventricular level as well as the twist, torsion, and θ_CL_ between basal and apical LV short-axis slices. A two-tailed *t*-test with unequal variance was used to test group-wise differences. Multiple comparisons were performed with Holm–Sidak post hoc correction. Multiple-regression analysis was used to test for correlations among biomarkers. A binomial logistic regression model assessed each biomarker’s ability to distinguish the following: (1) healthy volunteers vs. DMD patients, (2) healthy volunteers vs. LGE(−) DMD patients, and (3) LGE(−) DMD patients vs. LGE(+) DMD patients. **Results:** There was a significant impairment in the peak mid-wall E_cc_ [−17.0 ± 4.2% vs. −19.5 ± 1.9%, *p* < 7.8 × 10^−3^], peak LV twist (10.4 ± 4.3° vs. 15.6 ± 3.1°, *p* < 8.1 × 10^−4^), and peak LV torsion (2.03 ± 0.82°/mm vs. 2.8 ± 0.5°/mm, *p* < 2.6 × 10^−3^) of LGE(−) DMD patients when compared to healthy volunteers. There was a further significant reduction in the E_cc_, twist, torsion, and θ_CL_ for LGE(+) DMD patients when compared to LGE(−) DMD patients. In the LGE(+) DMD patients, age significantly correlated with LVEF (*r*^2^ = 0.42, *p* = 9 × 10^−3^), peak mid-wall E_cc_ (*r*^2^ = 0.27, *p* = 0.046), peak LV Twist (*r*^2^ = 0.24, *p* = 0.06), peak LV torsion (*r*^2^ = 0.28, *p* = 0.04), and peak LV θ_CL_ (*r*^2^ = 0.23, *p* = 0.07). In the LGE(−) DMD patients, only the peak mid-wall E_cc_ was significantly correlated with age (*r*^2^ = 0.25, *p* = 0.006). The peak LV twist outperformed the peak mid-wall LV E_cc_ and EF in distinguishing DMD patients from healthy volunteer groups (AUC = 0.88, 0.80, and 0.72), as well as in distinguishing LGE(−) DMD patients from healthy volunteers (AUC = 0.83, 0.74, and 0.62). The peak LV twist and peak mid-wall LV E_cc_ performed similarly in distinguishing the LGE(−) and LGE(+) DMD cohorts (AUC = 0.74, 0.77, and 0.79). **Conclusions:** The peak mid-wall LV E_cc_, peak LV twist, peak LV torsion, and peak LV θ_CL_ were significantly impaired in advance of the decreased LVEF and the development of focal myocardial fibrosis in boys with DMD and therefore were apparent prior to significant irreversible injury.

## 1. Introduction

Duchenne Muscular Dystrophy (DMD) is a prevalent fatal inherited genetic disorder that impacts 1:3802 to 1:6002 male births [1]. DMD inevitably results in progressive skeletal, respiratory, and cardiac muscle weakness—ultimately leading to the loss of ambulation and fatal respiratory or heart failure [2]. Owing to improvements in respiratory management, cardiomyopathy has emerged as a prevalent cause of morbidity and mortality in DMD. The onset and progression of cardiomyopathy is often subtle and highly variable in this cohort [3,4] due, in part, to the broad genotype. Consequently, there is a growing need for sensitive imaging biomarkers that identify and track the progression of cardiomyopathy in boys with DMD to improve patient care and evaluate the efficacy of novel therapeutics.

Current cardiac MRI exams are especially well suited to evaluation in the later stages of dilated cardiomyopathy associated with DMD, but the onset and speed of progression is highly variable from patient to patient [3,4]. Left ventricular ejection fraction (LVEF), as measured with cine MRI, has proven useful; boys with DMD that develop significant cardiac involvement eventually present with reduced LVEF [5]. An LVEF below 45% is a good predictor of fatal and nonfatal cardiac outcomes [6]. However, the time course for a specific patient to exhibit a measurable decline in LVEF is highly variable. Many boys with DMD have a normal (>55%) LVEF until late in the disease process, and small changes in LVEF are not useful in tracking myocardial disease progression. Alternatively, late gadolinium enhancement (LGE) imaging detects myocardial fibrosis. Positive LGE findings are frequently present in the mid and later stages of the DMD disease process and have been associated with systolic dysfunction [5,7,8,9]. Therefore, since boys with DMD are inherently at high risk for developing cardiac disease, monitoring is essential to appropriate patient care. Identifying boys with fomenting cardiac disease prior to obvious systolic dysfunction and assessing response to therapy is of great clinical importance.

Several newer cardiac MRI biomarkers have shown promise as earlier indicators of cardiac disease in boys with DMD, as compared to declines in LVEF and LGE positive (+) patients. For example, previous MRI tagging studies demonstrated that peak systolic mid-wall LV circumferential strain (E_cc_) can significantly distinguish DMD patients and healthy volunteers without significant differences in LVEF [9,10,11]. These results and others suggest that mid-ventricular E_cc_ is a sensitive MRI biomarker of cardiac systolic dysfunction before the appearance of reduced LVEF or positive LGE, but further validation is needed [12,13,14].

**Figure 1:**
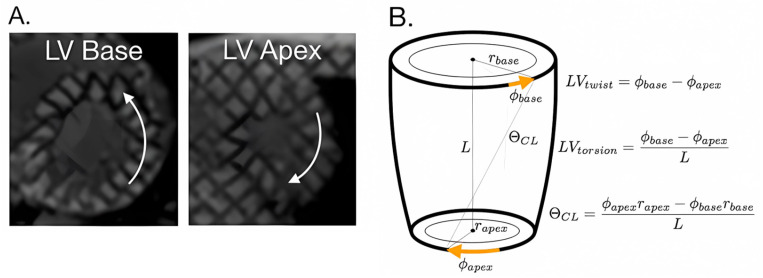
Representative tagged MRI images and measurement of LV rotational biomarkers: (**A**) Representative tagged MRI images. (**B**) Derivation of peak LV twist, torsion, and CL-shear angle (θ_CL_).

Cardiac MRI biomarkers of LV rotational mechanics, including the peak LV twist, peak LV torsion, and peak LV circumferential-longitudinal shear angle (θ_CL_) (Figure 1) could be earlier biomarkers of cardiac dysfunction in this cohort, and these metrics are readily assessed with MRI tagging. Reyhan et al. reported a significant decrease in the peak LV twist in five DMD patients compared to non-age-matched adult controls (*N* = 16) [15]. Previous studies have evaluated peak LV θ_CL_ in mitral regurgitation and DMD [15,16]. However, the peak LV θ_CL_ has been understudied in boys with DMD and may provide a measure of cardiac rotational mechanics independent of slice position or ventricular size [17]. Overall, biomarkers that measure the rotational mechanics of the heart may have value in observing early signs of cardiac disease in this cohort.

Our goals were (1) to characterize a spectrum of functional and rotational LV biomarkers in boys with DMD compared with healthy age-matched controls; and (2) to identify LV biomarkers of early cardiomyopathy in the absence of abnormal LVEF or LGE.

## 2. Methods

### 2.1. Study Enrollment

The local institutional review board approved this multi-center HIPAA compliant prospective study. All participants and/or legal guardians provided signed statements of informed consent. Pediatric patients with DMD (*N* = 43, all male, age = 13.8 ± 3.8 years) and age-matched healthy volunteers with no history of cardiac disease (*N* = 16, all male, age = 13.6 ± 2.9 years) were enrolled and underwent a cardiac MRI exam.

### 2.2. CMR Imaging

#### 2.2.1. CMR Protocol

Subjects were imaged at either 1.5 T or 3 T (Siemens Avanto or Skyra, Munich, Germany) according to the clinical availability of the scanners. The MRI exam consisted of localizers, short-axis tagging, contrast injection, cine imaging, and late gadolinium enhancement imaging.

#### 2.2.2. MRI Tagging

All subjects underwent the breath-held acquisition of basal, mid-ventricular, and apical LV short-axis grid-tagged MRI (spatial resolution = 1.4 × 1.4 × 8 mm^3^, TE = 2.12 ms, T_Res_ = 24–48 ms, 11–31 phases, grid tag spacing = 8 mm). The slice location was carefully chosen to provide consistent LV twist measurements. The basal slice was defined as the most basal short-axis slice location that contained the LV as a complete annular ring over the entire cardiac cycle, and the apical slice was defined as the most apical slice location in which the LV blood pool was a complete annular ring for the entire cardiac cycle. Re-acquiring images to meet these criteria was necessary in –30% of all cases and required less than one minute, thereby aiding the consistency of the LV twist measurements.

#### 2.2.3. Cine Imaging

A total of 26 patients were imaged post-contrast (0.1 mMol/kg of Gadobenate dimeglumine, MultiHance, Bracco Diagnostic Inc.: Milan, Italy) using a free-breathing retrospectively binned balanced steady-state free precession (bSSFP) cine sequence [18] (matrix = 192 × 120, spatial resolution = 1.4 × 1.4 mm^2^, TE = 1.2 ms, T_Res_ = 45.1–64.4 ms, flip angle = 40°, bandwidth = 930 Hz/Px). Breath-held bSSFP cine (matrix = 256 × 156, spatial resolution = 1.4 × 1.4 mm^2^, TE = 1.2 ms, T_Res_ = 28.1–45.1 ms, flip angle = 40–54°, bandwidth = 800–1300 Hz/Px) was used in 17 study participants when the free-breathing sequence was unavailable. A 16-channel body coil array was used to cover the entire LV from base to apex. Healthy volunteers (*N* = 16) were imaged with an identical free-breathing protocol without contrast. Images were acquired with full LV short-axis coverage (slice thickness = 8 mm, slice gap = 0 mm, number of slices = 10–16).

#### 2.2.4. LGE Imaging

Patients underwent post-contrast conventional breath-held LGE acquisitions with full LV short-axis coverage (*N* = 17, spatial resolution = 1.4 × 1.4 × 6.0 mm^3^, TE = 2.01 ms, T_Res_ = 750 ms) or free-breathing LGE imaging (*N* = 26, spatial resolution = 1.4 × 1.4 × 8.0 mm^3^, TE = 1.19 ms, T_Res_ = 904 ms, averages = 8) [19].

### 2.3. Post-Processing and Analysis

#### 2.3.1. Cine and LGE Analysis

A clinician (PR or AP) analyzed all cine and LGE images using commercial software (Circle CVI42, Circle Cardiovascular Imaging Inc., Calgary, AB, Canada) or Medis (Medis Cardiovascular Imaging, Leiden, The Netherlands). The following global LV functional parameters were estimated: LV-end systolic volume (LVESV) and LV-end diastolic volume (LVEDV); LV EF (LVEF), and LV mass (LVM); and each of the respective values indexed to the subject’s body surface area—LVESVi, LVEDVi, and LVMi. LGE images were assessed for the presence or absence of positive myocardial fibrosis and the location of a scar (when present) according to the AHA 17-segment model [20].

#### 2.3.2. Tagged MRI Analysis

Tagged MRI (Diagnosoft, Myocardial Solutions, Morrisville, NC, USA) was used to estimate the peak LV mid-wall Lagrangian circumferential strain (E_cc_) at the mid-ventricular level as well as the LV basal and apical angular rotation. The peak LV twist was defined as the difference in the peak angular rotation between the LV basal and LV apical tagged images (Figure 1) [17]. The peak LV torsion was computed from the peak LV twist normalized by the distance between the slices [17]. The peak LV CL-shear angle (θ_CL_) was computed as defined in Figure 1. To compute the Peak LV θ_CL_, estimates of apical and basal epicardial radii were made from averaging the length of the systolic semi-major and semi-minor elliptical axes from epicardial contours of the basal and apical slices.

### 2.4. Statistics

Statistical analysis was performed using Matlab (Mathworks, Natick, MA, USA). LV masses, volumes, and ejection fractions, as well as the peak mid-wall E_cc_, peak LV twist, peak LV torsion, and peak θ_CL_ were reported as the mean ± standard deviation and statistically compared between boys with DMD and healthy volunteers. In terms of normally distributed data, group-wise comparisons were performed with a two-tailed *t*-test with unequal variance. A Box–Cox transformation was applied to non-normally distributed data before comparison with a two-tailed *t*-test with unequal variance. The Holm–Sidak post hoc correction accounted for multiple comparisons.

Findings from the LGE data were also reported, and a sub-analysis stratified patients into “LGE negative (−) DMD” (no scar present) and “LGE(+) DMD” (more than one LGE(+) AHA segments) groups. LVEF and all functional and rotational mechanic parameters were compared between these groups utilizing the same method.

#### 2.4.1. Multiple-Regression Analysis

Correlations of all data with age and BMI, as well as relationships between LVEF and the peak mid-wall LV E_cc_, peak LV twist, peak LV torsion, and peak LV θ_CL_, were tested by multiple-regression analysis. A *p*-value < 0.05 was considered as a significant correlation.

#### 2.4.2. Binomial Logistic Regression

Each measured biomarker was analyzed with a binomial logistic regression model in the following distinguishing tasks: (1) healthy volunteers vs. a DMD population; (2) healthy volunteers vs. LGE(−) DMD patients; and (3) LGE(−) DMD patients vs. LGE(+) DMD patients. The predictive ability of each biomarker was displayed as receiver–operator curves (ROCs) and reported as the area under the curve (AUC). Finally, a generalized linear regression model incorporating the peak mid-wall LV E_cc_, peak LV twist, and LVEF was computed and compared to each biomarker individually using ROC analysis and the AUC.

## 3. Results

### 3.1. Demographics

Demographics for healthy volunteers, LGE(−) DMD, and LGE(+) DMD groups are summarized in Table 1. Overall, *N* = 16 patients (37%) were LGE(+). LGE(−) patients with DMD had a significantly increased BMI compared to healthy volunteers (24.4 ± 6.6 vs. 20.4 ± 5.8 kg/m^2^, *p* = 0.04), significantly decreased height (1.40 ± 0.17 vs. 1.64 ± 0.13 m, *p* = 5.5 × 10^−6^), and a significantly decreased BSA (1.34 ± 0.26 vs. 1.58 ± 0.35 m, *p* = 0.026) but were similar in other measures.

### 3.2. LV Volume and Function

Global measures of cardiac structure and function for patients with DMD and age-matched healthy volunteers are summarized in Table 2. A normal LVEF was defined as LVEF ≥ 55% [21]. Among the 27 LGE(−) patients with DMD, 9 exhibit early cardiomyopathy with an EF below 55%. However, as shown in Table 2, there are no statistically significant differences in LVEF, LVESV, LVED, LVEDVi, LVESVi, or LVMi between LGE(−) patients with DMD and healthy volunteers. A significant difference between LGE(−) patients with DMD and healthy volunteers was found in LVM (65.6 ± 22.0 vs. 66.7 ± 22.7 g^2^, *p* < 0.04). There is no significant difference in LVEF between LGE(−) patients with DMD and healthy volunteers (54.5 ± 6.8% vs. 57.7 ± 4.0%, *p* = 0.06). But LGE(+) patients with DMD had significantly impaired LVEF comparing to LGE(−) patients with DMD (44.6 ± 11.2% vs. 54.5 ± 6.8%, *p* = 0.06). No significant differences were observed between other measures.

### 3.3. Peak Mid-Wall LV E_cc_ and Peak LV Rotational Biomarkers

The peak mid-wall LV E_cc_, peak LV twist, peak LV torsion, and peak LV θ_CL_ were compared among boys with DMD and age-matched healthy volunteers (Table 3). There was a significant difference between patients with DMD and volunteers in peak mid-wall E_cc_ (−15.9 ± 4.5% vs. −19.5 ± 1.9%, *p* < 3.9 × 10^−4^), peak LV twist (9.0 ± 4.7° vs. 15.6 ± 3.1°, *p* < 1.1 × 10^−4^), peak LV torsion (1.7 ± 0.9 °/mm vs. 2.8 ± 0.50 °/mm, *p* < 1.1 × 10^−4^), and peak LV θ_CL_ (5.2 ± 2.5° vs. 7.04 ± 3.5°, *p* < 2.1 × 10^−3^).

Comparing healthy volunteers with LGE negative patients with DMD, a statistically significant impairment was observed in the peak mid-wall E_cc_ (−17.0 ± 4.2% vs. −19.5 ± 1.9%, *p* < 7.8 × 10^−3^), peak LV twist (10.4 ± 4.3° vs. 15.6 ± 3.1°, *p* < 8.1 × 10^−4^), and peak LV torsion (2.03 ± 0.82 °/mm vs. 2.8 ± 0.5 °/mm, *p* < 2.6 × 10^−3^).

When comparing between LGE(−) and LGE(+) patients with DMD, there was a further significant and progressive impairment in all cardiac MRI biomarkers.

### 3.4. Multiple-Regression Analysis

**Figure 2:**
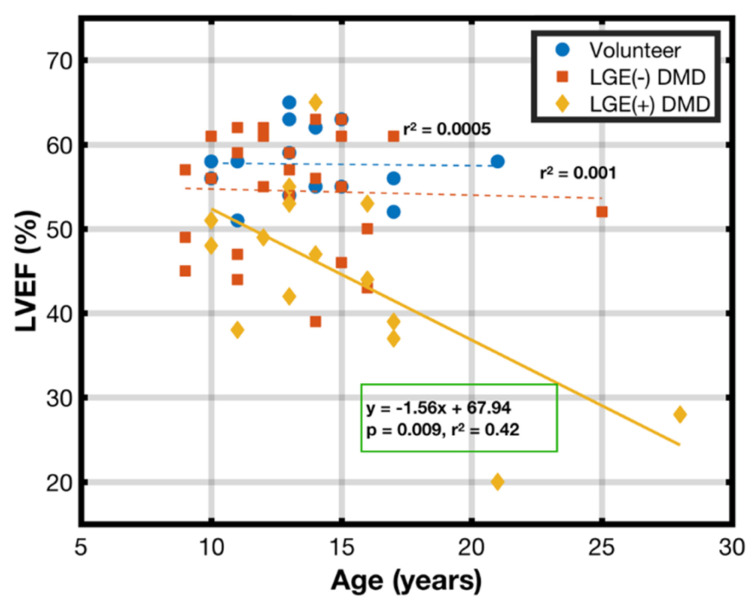
Age vs. LV ejection fraction (LVEF) in volunteers (blue circles), LGE(−) patients with DMD (orange squares), and LGE(+) patients with DMD (yellow diamonds). Only LGE(+) patients with DMD have a significant negative correlation. There is no significant relationship between age and LVEF for healthy volunteers and LGE(−) patients with DMD.

Figure 2 shows the age-dependent correlations of LVEF for each of the three populations: healthy volunteers, LGE(−) DMD, and LGE(+) DMD. No relationship between age and LVEF was observed for either the healthy volunteers or the LGE(−) DMD patients, but a significant negative correlation (*r*^2^ = 0.42, *p* = 9 × 10^−3^) was observed for the LGE(+) DMD patients, corresponding to a decrease in the EF of 1.56% per year over the population.

**Figure 3:**
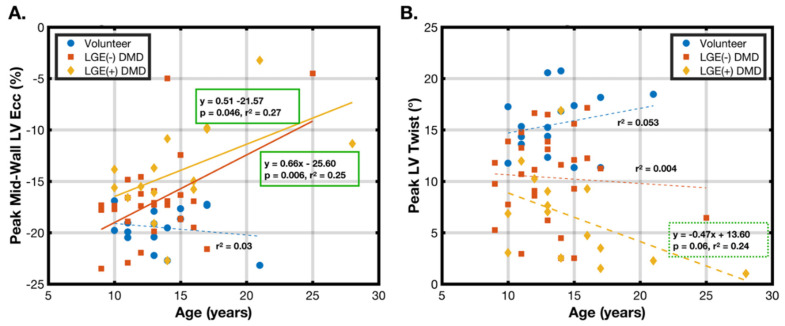
Age vs. the peak mid-wall LV Ecc and peak LV twist in healthy volunteers (blue circle), LGE(−) DMD boys (orange square), and LGE(+) DMD boys (yellow diamonds): (**A**) peak mid-wall LV Ecc vs. age; and (**B**) peak LV twist vs. age. A significant correlation is identified with multiple linear regression (solid lines), and non-significant correlations are shown (dashed lines) for completeness. The peak mid-wall LV Ecc and peak LV twist decrease moderately with age in the LGE(+) DMD patients. The peak mid-wall LV Ecc also decreases with age in the LGE(−) DMD patients. There is no correlation with age in the healthy volunteer group for either measure or for the peak LV twist in the LGE(−) DMD patients.

Figure 3 demonstrates age-dependent correlations of the peak mid-wall E_cc_ (Figure 3A) and peak LV twist (Figure 3B) for the healthy volunteers, LGE(−) DMD, and LGE(+) DMD groups. For the healthy volunteers, there was no age dependence with the peak mid-wall E_cc_ (*r*^2^ = 0.03, *p* = N.S.) or peak LV twist (*r*^2^ = 0.05, *p* = N.S). In the LGE(+) DMD patients, age correlated with the E_cc_ (*r*^2^ = 0.27, *p* = 0.046) and peak LV twist (*r*^2^ = 0.24, *p* = 0.06). This corresponds to a 0.5% (strain) decrease in the peak mid-wall E_cc_ and a 0.5° decrease in peak LV twist per year across the cohort. In the LGE(−) DMD patients, no correlations were found between peak LV twist and age (*r*^2^ = 0.004, *p* = N.S.), but the peak mid-wall LV E_cc_ was correlated with age (*r*^2^ = 0.25, *p* = 0.006), corresponding to an overall decrease of 0.7% (strain) per year across the cohort.

Age correlations for peak LV torsion and peak LV θ_CL_ are not shown but closely echo the peak LV twist results. Peak LV torsion and age correlated only in the LGE(+) patients (*r*^2^ = 0.28, *p* = 0.04) corresponded to a change of −0.10 °/mm/year. The peak LV θ_CL_ also only depended on age in the LGE+ patients (*r*^2^ = 0.23, *p* = 0.07), corresponding to a decrease of −0.24 °/year. No correlation existed between any measured cardiac MRI biomarker and BMI in any group.

**Figure 4:**
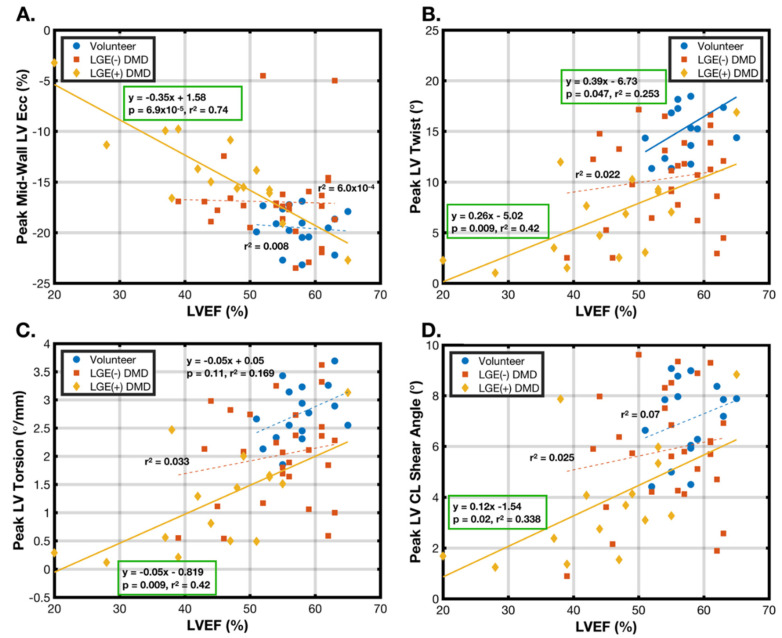
Peak mid-wall LV E_cc_ and peak LV rotational biomarkers as functions of LVEF in healthy volunteers (blue circles), LGE(−) DMD boys (orange squares), and LGE(+) DMD boys (yellow diamonds): (**A**) peak mid-wall LV E_cc_; (**B**) peak LV twist; (**C**) peak LV torsion; and (**D**) peak LV θ_CL_. Significant correlations are identified with multiple linear regression (solid lines). Non-significant correlations are shown (dashed lines) for completeness. All cardiac MRI biomarkers are significantly correlated with LVEF for the LGE(+) patients with DMD. There is also a significant correlation between the peak LV twist and LVEF in the healthy volunteers. For all other groups, and, particularly, the LGE(−) DMD patients, these cardiac MRI biomarkers are uncorrelated with EF.

Figure 4 demonstrates the peak mid-wall LV E_cc_ (Figure 4A), peak LV twist (Figure 4B), peak LV torsion (Figure 4C), and peak LV θ_CL_ (Figure 4D) as functions of LVEF in healthy volunteers, LGE(−) DMD boys, and LGE(+) DMD boys. Most notably, the peak LV twist and LVEF were significantly correlated in the healthy volunteers (*r*^2^ = 0.25, *p* = 0.047) and in the LGE(+) DMD patients (*r*^2^ = 0.42, *p* = 0.009). No similar relationship was observed in the LGE(−) DMD patients (*r*^2^ = 0.02, *p* = 0.45). There was, however, a very strong relationship between the peak mid-wall E_cc_ and EF in the LGE(+) DMD patients (*r*^2^ = 0.72, *p* = 6.9 × 10^−5^) but no relationship for the other two populations. Peak LV torsion and peak LV θ_CL_ results generally echoed the peak LV twist results. For LGE(+) patients, peak LV torsion correlated well with LVEF (*r*^2^ = 0.42, *p* = 0.009) as did the peak LV θ_CL_ (*r*^2^ = 0.338, *p* = 0.020). There was a weak, non-significant correlation between LVEF and peak LV torsion in the healthy volunteers (*r*^2^ = 0.17, *p* = 0.11). No correlations existed with LVEF in the LGE(−) DMD patients.

Importantly, 61% of the of LGE(−) DMD patients presented with clinically normal LVEF (>55%). Of LGE(−) patients with normal LVEF, 46% had peak LV twist values >1 standard deviation less than the mean for healthy volunteers (<12.5° peak LV twist). A total of 32% had peak mid-wall LV E_cc_ values >1 standard deviation less than that for healthy volunteers (<−17.6%).

### 3.5. Binomial Logistic Regression

**Figure 5:**
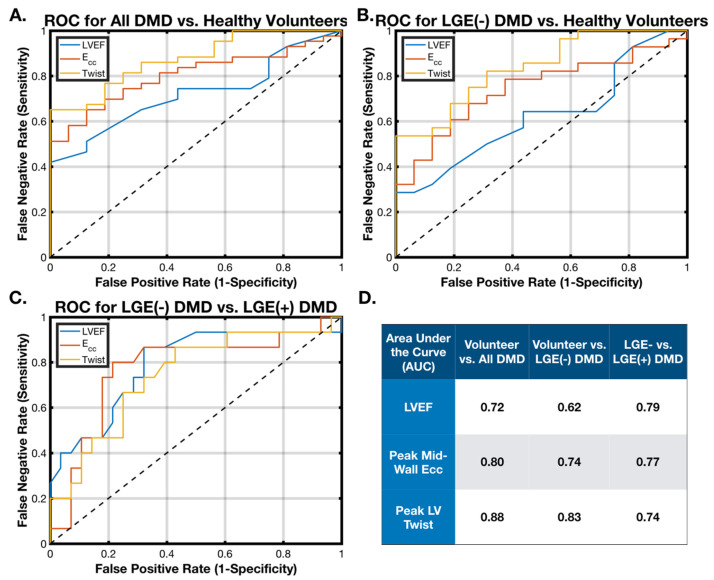
ROCs for the binomial logistic regression analysis differentiating (**A**) all patients with DMD vs. healthy volunteers, (**B**) LGE(−) patients with DMD vs. healthy volunteers, and (**C**) LGE(+) vs. LGE(−) DMD patients. (**D**) The area under the ROC curve for the peak LV twist, peak mid-wall E_cc_, and EF for each comparison. The peak LV twist (followed by E_cc_) is the best biomarker to differentiate both all patients with DMD from healthy volunteers and LGE(−) patients with DMD from healthy volunteers. All cardiac MRI biomarkers are effective at differentiating patients with DMD and advanced cardiac disease (LGE(+)) from DMD patients without advanced cardiac disease (LGE(−)).

Figure 5 shows the results of the logistic regression comparing the ability of LVEF, the peak LV twist, and the peak mid-wall LV E_cc_ to distinguish between the healthy volunteers and DMD patients (Figure 5A), the healthy volunteers and LGE(−) DMD patients (Figure 5B), and the LGE(−) DMD and LGE(+) DMD patients (Figure 5C). The corresponding AUC values for each analysis from Figure 5A–C are summarized in Figure 5D. The peak LV twist was best able to distinguish between the DMD and healthy volunteer groups, followed by the peak mid-wall LV E_cc_ and EF (AUC = 0.88, 0.80, and 0.72), as well as between the healthy vs. LGE(−) groups (AUC = 0.83, 0.74, and 0.62). All three methods were roughly equivalent in distinguishing the LGE(−) and LGE(+) DMD groups (AUC = 0.74, 0.77, and 0.79). Not shown are the ROCs and generally lower AUC values for peak LV torsion and the peak LV θ_CL_ for distinguishing between healthy volunteers and all patients with DMD (0.76 and 0.65), between healthy volunteers and LGE(−) patients with DMD (0.83 and 0.73), and between LGE(+) and LGE(−) patients with DMD (0.76 and 0.75).

**Figure 6:**
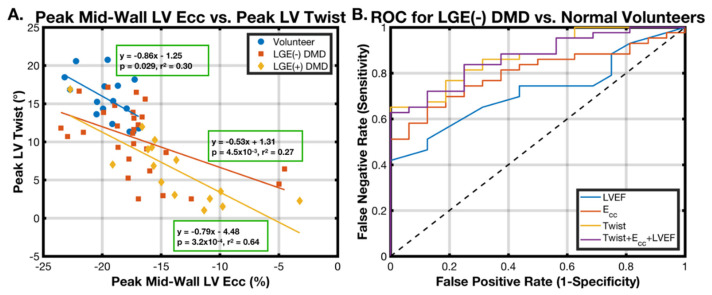
Correlation analysis and receiver operator characteristic (ROC) curves: (**A**) The correlations between the peak mid-wall LV E_cc_ and peak LV twist. Significant correlations identified with multiple linear regression are shown as solid lines. The peak LV twist and peak mid-wall E_cc_ are highly correlated in the LGE(+) DMD patients and moderately correlated in the LGE(−) DMD and healthy volunteer groups. (**B**) ROC analysis of the binary logistic regression generalized linear model incorporating the combination of the peak mid-wall LV E_cc_, peak LV twist, and LVEF together, and the logistic regression for each shown separately. Only the peak LV twist was a significant co-factor in the combined model, and the AUC of the combined model was marginally less than that of the peak LV twist alone (0.830 vs. 0.837).

Correlation analysis was used to determine whether a combination of several cardiac MRI biomarkers (peak mid-wall E_cc_, peak LV twist, and LVEF) can outperform the peak LV twist for distinguishing between healthy volunteers and LGE(−) DMD patients. Figure 6A depicts the correlations between the peak mid-wall LV E_cc_ and peak LV twist, and Figure 6B shows the ROC analysis of the generalized linear model incorporating the peak mid-wall LV E_cc_, peak LV twist, and LVEF together, and each biomarker modeled separately. The peak mid-wall LV E_cc_ and peak LV twist were significantly correlated in healthy, LGE(−) DMD, and LGE(+) DMD groups, respectively (*p* = 0.029, 4.5 × 10^−3^, and 3.2 × 10^−4^; *r*^2^ = 0.30, 0.27, and 0.64). Only the peak LV twist was a significant cofactor in the combined model, and the AUC of the combined model was marginally less than that of the peak LV twist alone (0.830 vs. 0.837), indicating that the use of the peak mid-wall LV E_cc_ or LVEF in combination with the peak LV twist does not improve the predictive ability of the peak LV twist alone to distinguish between LGE(−) DMD patients and healthy volunteers.

## 4. Discussion

A statistically significant impairment was observed in the peak LV twist, peak LV torsion, peak LV θ_CL_, and peak mid-wall LV E_cc_ of DMD patients, which precede decreases in LVEF or the presence of scarring as measured by LGE. All cardiac MRI biomarkers including LVEF effectively distinguished DMD patients from age-matched healthy volunteers, as well as between LGE(+) DMD patients and LGE(−) DMD patients. However, only the peak LV twist, peak LV torsion, and peak mid-wall LV E_cc_ significantly differentiated the healthy volunteers from the LGE(−) DMD patients (Table 4 and Figure 5D).

Distinguishing LGE(−) DMD patients from age-matched healthy volunteers offers substantial evidence that these cardiac MRI biomarkers reveal an early indication of cardiac dysfunction. Cardiac pathology in DMD is complex, heterogeneous, and does not uniformly impact cardiac function in all patients. Consequently, some patients who appear to have normal cardiac function (>55% LVEF and LGE(−)) will go on to develop signs of significant cardiac disease (<55% EF and LGE(+)) later in life, but others may remain relatively normal for prolonged periods. LVEF (and to a somewhat lesser extent, the mid-wall LV E_cc_, peak LV twist, and peak LV torsion) are well correlated with age in LGE(+) patients with DMD (Figure 2 and Figure 3), suggesting that, once overt signs of cardiac disease develop, all of these biomarkers continue to decline. This cohort progression is indicated in Table 4, highlighting significant differences in all measured biomarkers between healthy volunteers and patients with DMD. However, only the peak mid-wall LV E_cc_, peak LV twist, and peak LV torsion are significantly different between the healthy volunteers and LGE(−) DMD patients. Furthermore, the peak mid-wall E_cc_ and peak LV twist provide physiologically grounded evidence of reduced function (Figure 4A,B) in many (32% and 46%, respectively) of the 61% of DMD patients who were LGE(−) with normal EF.

Importantly, Figure 4B shows that LVEF and the peak LV twist are well correlated with one another in both the healthy and LGE(+) groups, which are composed of boys with either normal or reduced systolic function, respectively. The LGE(−) DMD patients, who are either earlier in the presentation or who may ultimately be less affected, show no correlation between LVEF and the peak LV twist. We conclude that the peak LV twist is potentially able to identify early changes in LV function that are not apparent in measures of LVEF.

We also evaluated an exponential regression model for the multiple-regression analysis as it may better detect rapid changes and found results entirely consistent with our linear regression model.

Our mid-wall E_cc_ strain results are consistent with several prior reports [9,10,11]. In a cohort of patients with DMD (*N* = 70) and healthy volunteers (*N* = 16), Hor et al. demonstrated that both young patients (<10 years) and age-matched patients (>10 years) with normal LVEF had significantly reduced mid-ventricular E_cc_ compared to healthy volunteers [9] and reported strain values consistent with those found in this study. Ashford et al. reported significantly reduced (−17 ± 3% vs. −15 ± 2%) mid-ventricular and basal E_cc_ in a cohort of patients with DMD (*N* = 13, 10.6 ± 3.0 years) compared to healthy volunteers (*N* = 9, 11.1 ± 2.5 years) despite normal LV volumes and LVEF [10].

However, the most effective predictor differentiating the LGE(−) DMD patients from healthy volunteers (Figure 5D) was the LV twist, which has not previously been reported in this context. The mean LV twist values observed in patients with DMD (9.0 ± 4.7°) were consistent with a prior report in five patients by Reyhan et al. (10.5 ± 3.6°) [15]. The LV twist had the highest AUC of any biomarker measured between LGE(−) DMD boys and healthy volunteers, and, when the peak mid-wall LV E_cc_ and LVEF were also incorporated into a generalized linear model, the predictive ability did not improve (Figure 6B). In fact, the peak LV twist was the only significant cofactor in this combined model. Taken together with the peak LV twist’s high correlation with the peak mid-wall LV E_cc_ (Figure 6A), the data suggest that the peak LV twist is the best and only biomarker needed to differentiate these groups and that the addition of LVEF and the peak mid-wall LV E_cc_ fails to provide additional useful information. Peak LV θ_CL_ results were also similar to those measured by Reyhan et al. in five DMD patients (5.3 ± 2.5°) vs. sixteen healthy adult controls (5.9 ± 1.7°) but are reported here in a large DMD cohort with distinctions between LGE(+) and LGE(−) along with age-matched healthy volunteers for the first time.

Both the peak LV twist and peak mid-wall LV E_cc_ are linked to underlying cardiomyocyte performance, and observing reductions in their performance may capture an early “snapshot” of the transition between normal cardiac performance, and the later overt effects of cardiomyopathy in DMD as measured by LGE(+) and reduced LVEF. This preliminary information is essential for assessing new therapies and initiating early treatment for cardiac symptoms in DMD.

Previous reports made careful arguments for the standardization of biomarkers of the LV rotation. We use the terminology defined by Young et al. and demonstrated in Figure 1 [17]. However, for standardization and cross-validation purposes, it is important to note that other works chose other definitions for these terms. While we agree with Rüssel et al. that the standardization of terms is important and that the peak LV θ_CL_ is the preferred metric to do so in theory [22], no currently available clinical analysis software is able to provide this metric without significant post-processing time and the introduction of several subjective steps into the overall measurement. On the other hand, the peak LV twist is readily assessed from tagged MRI images using Matlab which is a FDA-approved and clinically available tool. Furthermore, peak LV torsion and peak LV θ_CL_ results closely echo those of the peak LV twist results but modestly underperform the peak LV twist in terms of predictive ability as defined by the AUC of the ROCs in Figure 5. These results indicate that careful attention to slice placement during image acquisition permits meaningful measurements of the peak LV twist that distinguishes between healthy volunteers, LGE(+) DMD patients, and LGE(−) DMD patients.

### Limitations

Our study’s limited sample size did not allow for *n*-fold cross validation or an independent training test set to characterize this rare disease, instead relying on the area under the curve of our logistic models to distinguish the best biomarker for differentiating the DMD patients and healthy volunteers. While this study is limited by the use of single-time point evaluations of boys with DMD, the results suggest a progression in the development of cardiac disease not well characterized by classically used biomarkers such as LVEF and LGE(+). The reliability of the LV E_cc_, twist, torsion, and θ_CL_ as biomarkers may be impacted by measurement variability introduced by the user’s skill or scanner variations. This study has a limitation due to the absence of detailed phenotypic information, which could have provided deeper insights into patient characteristics and variability. This study is also limited by the lack of assessment of the motor performance of DMD patients. But a previous study reported that a 6 min walk test (6MWT) and North Star Ambulatory Assessment (NSAA), which assessed the motor performance of DMD patients, have no correlation with the global circumferential strain but in longitudinal strain [23]. On-going work will enable the evaluation of within-subject longitudinal changes in these same cardiac MRI biomarkers. The correlation of ambulation and cardiac medication with LVEF, peak mid-wall LV E_cc_, and peak LV rotational biomarkers should be considered for future work. Future studies could also look into the severity of LGE and how it relates to LVEF, peak mid-wall LV E_cc_, and peak LV rotational biomarkers.

## 5. Conclusions

Decreases in functional (peak mid-wall LV E_cc_) and rotational (peak LV twist, peak LV torsion, and peak LV θ_CL_) cardiac MRI biomarkers precede larger-scale changes in LVEF and the development of myocardial fibrosis in boys with DMD. Of all the biomarkers evaluated, the peak LV twist was best able to distinguish between age-matched healthy volunteers and LGE(−) DMD patients, followed closely by the peak mid-wall LV E_cc_. Both of these measurements are readily implemented into a clinical MRI acquisition and post-processing workflows. These could be important tools for the clinical evaluation of early cardiac dysfunction in DMD, as the consequence of novel therapeutics and disease modifying therapies.

## Figures and Tables

**Table 1:** Demographics of DMD patients and age-matched healthy volunteers.

Patient Demographics	LGE(+) DMD *N* = 16	LGE(−) DMD *N* = 27	Healthy Volunteers *N* = 16
Age (years)	15.0 ± 4.5	13.1 ± 3.2	13.6 ± 2.8
Male (%)	100%	100%	100%
Height (cm)	1.44 ± 0.13	1.40 ± 0.17 ^†^	1.64 ± 0.13 ^†^
Weight (kg)	55.14 ± 12.89	47.26 ± 14.0	56.4 ± 23.0
BMI (kg/m^2^)	26.3 ± 5.1	24.4 ± 6.6 ^†^	20.4 ± 5.8 ^†^
BSA (m^2^)	1.48 ± 0.23	1.34 ± 0.26 ^†^	1.58 ± 0.35 ^†^

Data are reported as median ± standard deviation; BMI, body mass index; BSA, body surface area. ^†^ *p* < 0.05 between LGE(−) DMD patients and healthy volunteers.

**Table 2:** Global measures of cardiac structure and function derived from cine imaging in DMD patients and age-matched healthy volunteers.

Cardiac Function	LGE(+) DMD *N* = 16	LGE(−) DMD *N* = 27	Healthy Volunteers *N* = 16
LVEF (%)	44.6 ± 11.2 *	54.5 ± 6.8 *	57.7 ± 4.0
LVEDVi (mL/m^2^)	95.7 ± 38.8	87.5 ± 38.4	52.9 ± 6.0
LVESVi (mL/m^2^)	55.9 ± 34.9	39.5 ± 18.4	22.3 ± 3.3
LVMi (g/m^2^)	43.9 ± 11.2	39.6 ± 11.7	41.5 ± 7.8
LVEDV (mL)	140.8 ± 60.3	117.7 ± 65.0	143.9 ± 33.2
LVESV (mL)	82.1 ± 52.8	53.3 ± 31.2	60.7 ± 15.8
LVM (g)	65.6 ± 22.0	52.6 ± 16.4 ^†^	66.7 ± 22.7 ^†^

* *p* < 0.05 between LGE(+) and LGE(−) DMD patients with post hoc correction. ^†^ *p* < 0.05 between LGE(−) DMD patients and healthy volunteers.

**Table 3:** Cardiac MRI biomarker of LV rotational mechanics for DMD patients and age-matched healthy volunteers.

Rotational Mechanics	DMD Patients *N* = 43	Healthy Volunteers *N* = 16	*p*-Value
Peak mid-wall E_cc_ * (%)	−15.9 ± 4.5	−19.5 ± 1.9	3.9 × 10^−4^
Peak LV Twist * (°)	9.0 ± 4.7	15.6 ± 3.1	3.4 × 10^−6^
Peak LV Torsion * (°/mm)	1.7 ± 0.9	2.8 ± 0.5	1.1 × 10^−4^
Peak LV θ_CL_ * (°)	5.3 ± 2.5	7.04 ± 3.5	6.8 × 10^−3^

Significant differences existed between each measured biomarker. * *p*-value ≤ 0.05 is significant.

**Table 4:** Comparison of several biomarkers for LGE(+) DMD patients and LGE(−) DMD patients when compared to healthy volunteers, respectively.

Cohorts	LGE(+) DMD *N* = 16	LGE(−) DMD *N* = 27	Healthy Volunteers *N* = 16
Peak mid-wall E_cc_* (%)	−13.9 ± 4.5 *	−17.0 ± 4.2 *^,†^	−19.5 ± 1.9 ^†^
Peak LV Twist * (°)	6.5 ± 4.7 *	10.4 ± 4.3 *^,†^	15.6 ± 3.1 ^†^
Peak LV Torsion * (°/mm)	1.2 ± 0.9 *	2.0 ± 0.8 *^,†^	2.8 ± 0.5 ^†^
Peak LV θ_CL_ * (°)	3.8 ± 2.3 *	5.9 ± 2.4 *	7.0 ± 1.6

* *p* < 0.05 between LGE(+) and LGE(−) DMD patients with post hoc correction. ^†^ *p* < 0.05 between LGE(−) DMD patients and healthy volunteers.

## Data Availability

The datasets used and/or analyzed during the current study are available from the corresponding author on reasonable request.
